# Identification of mutations through dominant screening for obesity using C57BL/6 substrains

**DOI:** 10.1038/srep32453

**Published:** 2016-09-02

**Authors:** Mohammad Sarowar Hossain, Fuyuki Asano, Tomoyuki Fujiyama, Chika Miyoshi, Makito Sato, Aya Ikkyu, Satomi Kanno, Noriko Hotta, Miyo Kakizaki, Takato Honda, Staci J. Kim, Haruna Komiya, Ikuo Miura, Tomohiro Suzuki, Kimio Kobayashi, Hideki Kaneda, Vivek Kumar, Joseph S. Takahashi, Shigeharu Wakana, Hiromasa Funato, Masashi Yanagisawa

**Affiliations:** 1 International Institute for Integrative Sleep Medicine (WPI-IIIS), University of Tsukuba, 1-1-1 Tennodai, Tsukuba, Ibaraki 305-8575, Japan; 2 Ph.D. Program in Human Biology, School of Integrative and Global Majors, University of Tsukuba, 1-1-1 Tennodai, Tsukuba Ibaraki, 305-8575, Japan; 3 Technology and Development Team for Mouse Phenotype Analysis, RIKEN BioResource Center, Tsukuba, Ibaraki 305-0074, Japan; 4 Department of Neuroscience, University of Texas Southwestern Medical Center at Dallas, Texas 75390, USA; 5 The Jackson Laboratory, Bar Harbor, ME 04609, USA; 6 Howard Hughes Medical Institute, University of Texas Southwestern Medical Center at Dallas, Taxas, 75390, USA; 7 Department of Anatomy, Toho University Faculty of Medicine, Tokyo 143-8540, Japan; 8 Department of Molecular Genetics, University of Texas Southwestern Medical Center at Dallas, Texas 75390, USA

## Abstract

The discovery of leptin substantiated the usefulness of a forward genetic approach in elucidating the molecular network regulating energy metabolism. However, no successful dominant screening for obesity has been reported, which may be due to the influence of quantitative trait loci between the screening and counter strains and the low fertility of obese mice. Here, we performed a dominant screening for obesity using C57BL/6 substrains, C57BL/6J and C57BL/6N, with the routine use of *in vitro* fertilization. The screening of more than 5000 mutagenized mice established two obese pedigrees in which single nucleotide substitutions in *Mc4r* and *Sim1* genes were identified through whole-exome sequencing. The mutation in the *Mc4r* gene produces a premature stop codon, and the mutant SIM1 protein lacks transcriptional activity, showing that the haploinsufficiency of SIM1 and MC4R results in obesity. We further examined the hypothalamic neuropeptide expressions in the mutant pedigrees and mice with diet-induced obesity, which showed that each obesity mouse model has distinct neuropeptide expression profiles. This forward genetic screening scheme is useful and applicable to any research field in which mouse models work.

Obesity results from a sustained positive energy balance in which the accumulated energy is stored as fat, mainly in adipose tissues. Although an animal’s energy balance is regulated in a homeostatic manner to keep body weight stable, the mechanism for suppressing overweight is not tightly regulated, and the obesity pandemic has become one of the most severe global health problems due to the availability of food that is cheap and has a high caloric content.

After the seminal discovery of leptin and the long form of the leptin receptor (LepRb) through forward genetic studies on recessive obese mouse pedigrees, *ob/ob* and *db/db*^1^, the hypothalamus has been recognized as an important brain region involved in integrating the negative feedback signals received from peripheral adipose tissues via leptin to modulate food intake and energy expenditure^2^. In the hypothalamic arcuate nucleus, agouti-related peptide (AGRP)-expressing neurons promote food intake and suppress energy expenditure, whereas proopiomelanocortin (POMC)-expressing neurons suppress food intake and enhance energy expenditure. In response to leptin, AGRP neurons are inhibited and POMC neurons are activated, which together suppress food intake and enhance energy expenditure, resulting in a decrease in body fat content. Major downstream targets of AGRP and POMC neurons are the melanocortin receptor 4 (MC4R)-expressing neurons of the paraventricular nucleus (PVN), where the axons of POMC neurons secrete α-melanocyte-stimulating hormone (α-MSH), which is an endogenous ligand for MC4R^3^,^4^. Conversely, AGRP works as an inverse agonist on MC4R^4^. In the opposite direction of signaling from the arcuate nucleus to the PVN, pituitary adenylate cyclase-activating polypeptide (PACAP, also known as ADCYAP1)- and thyrotropin-releasing hormone (TRH)-expressing neurons of the PVN provide monosynaptic excitatory inputs to AGRP neurons that enhance feeding behavior^5^, suggesting that the arcuate nucleus and PVN form a reciprocal circuit to drive food intake. The crucial role of PVN neurons in body weight regulation has been further verified by the development of obesity after the ablation of SIM1-positive PVN neurons in adult mice^6^. The lateral hypothalamic area (LHA) express orexin (also known as hypocretin), which is associated with energy metabolism and resistance to diet-induced obesity^7^,^8^. In contrast, a deficiency in another LHA-specific neuropeptide, melanin-concentrating hormone (MCH), causes mice to become leaner as a result of increased oxygen consumption^9^.

The successful cloning of the *obese* gene substantiated the scientific significance of a forward genetic approach, which is free from any hypothesis and is conducted in an unbiased way. In fact, in parallel with circuit-based research on energy metabolism as mentioned above, forward genetic studies have confirmed the recessive inheritance of obesity-causing mutations in the *leptin*^10^, *leptin receptor*^11^ and *Mc4r*^12^ genes. However, a successful screening for dominant heritable traits related to obesity has not yet been reported.

A major obstacle in this screening is the quantitative trait loci (QTLs) between the screening strain treated with a chemical mutagen, ethylnitrosourea (ENU), and the counter strain used for the backcross. To locate the chromosomal region associated with obesity, the mutated mice need to be crossed with other strains of mice to make N2 generation mice, which are used for linkage and haplotype analyses. However, each inbred mouse strain has a characteristic profile of energy metabolism and body weight regulation^13^ under the influence of QTLs^14^. The different metabolic effects of different QTLs between the mutagenized and backcross strains may confound the mapping of the mutant locus and lower the statistical power to detect the mutant locus based on LOD score^15^. Ideally, a mutagenized strain and a counter strain would be identical in their genomic DNA except for 4–6 single nucleotide polymorphisms (SNPs) per chromosome. However, because of available polymorphic markers such as microsatellites and SNPs between inbred mouse strains, ENU-treated C57BL/6J (B6J) mice have been crossed with DBA/2J^16^, BALB/c^10^ or C3H/He^12^,^17^ mice. These inbred strains have more than 5,000,000 genetic variations relative to the B6J strain, reflecting the different history and origin of each strain^18^. In contrast, the C57BL/6N (B6N) strain, a B6 substrain, was originally established from a group of B6J mice shipped to the NIH in 1951 and has approximately 17,000 genetic variants relative to B6J mice with only 108 non-synonymous coding changes^19^. The entire list of single nucleotide polymorphisms (SNPs) between the B6J and B6N mice enables us to use these substrains as a screening stain and counter strain, respectively.

Another major obstacle to dominant obesity screening is the difficulty in maintaining obese pedigrees through natural mating between obese male and female mice because obese mice are often infertile^20^,^21^. Thus, the routine use of *in vitro* fertilization is necessary to establish and maintain a dominant obese pedigree.

Here, we conducted dominant screening for obesity using B6J and B6N mice, with the routine use of *in vitro* fertilization for offspring production. Then, we identified mutations in two dominant obese pedigrees using a candidate gene approach and whole-exome sequencing.

## Results

### Establishment of obese pedigrees

**Figure 1:**
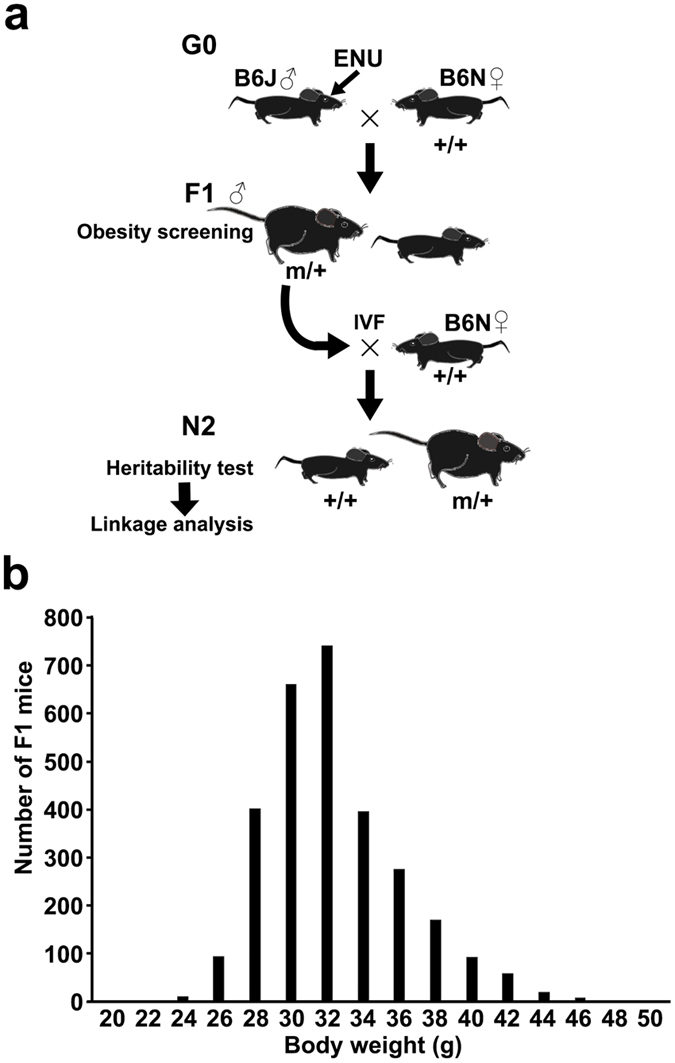
Obesity screening of randomly mutagenized mice. (**a**) C57BL/6J (B6J) males were treated with ethylnitrosourea (ENU) via intraperitoneal injections and crossed with C57BL/6N (B6N) females to generate the offspring (F1 mice). The F1 mice were screened for obesity. Sperm cells from obese mice were used for the *in vitro* fertilization of wild-type B6N eggs to obtain the N2 mice. The N2 progeny were examined for heritable obesity. If the pedigree showed heritable obesity, N2 mice were further used for a linkage analysis. (**b**) The histogram shows the body weight of all F1 mice screened at 18 weeks old. n = 2940.

In parallel with the sleep/wakefulness screening of randomly mutagenized B6J/B6N F1 male mice, which were the offspring of a cross between ethylnitrosourea (ENU)-treated B6J male mice and wild-type B6N female mice, we examined the same mice for obesity (Fig. 1a). The body weight distribution of mutagenized F1 mice (n = 2940) at 18 weeks old was skewed toward overweight (mean = 33.3 g, standard deviation = 3.76 g and skewness = 0.85, Fig. 1b). In addition to the F1 mice, we screened 2500 mutagenized G1 mice, which were the offspring of a cross between ethylnitrosourea (ENU)-treated B6J male mice and wild-type B6J female mice.

Because there have been no successful dominant screenings for obesity, we were not able to establish reliable criteria for selecting obese mice that have a mutation causing dominant obesity. In our screening, we therefore selected obese mice at several ages to include early-onset and late-onset obesity, increasing the possibility of establishing heritable obese pedigrees. We selected the five heaviest male mice (body weight: 46 g, 46 g, 48 g, 48 g and 51 g) at 18 weeks old and the five heaviest male mice (body weight: 50 g, 51 g, 51 g, 51 g and 53 g) at 28 weeks old. We also selected two obese mice with high blood glucose levels (body weight and blood glucose: 52 g and 286 mg/dl, 53 g and 266 mg/dl) at 32 weeks old. In addition, we selected one mouse that showed severe early-onset obesity, weighing 47 g at the age of 10 weeks. In total, 13 obese mice were used to produce progeny via *in vitro* fertilization using eggs from C57BL/6N mice (Fig. 1a). When at least 30% of the progeny developed obesity similar to the founder mouse, we considered the obesity phenotype to be heritable. This criterion was set because we were looking for gene mutations that reproducibly cause dominant obesity. Among the 13 pedigrees, two pedigrees, *Obese-13* and *Obese-10,* showed heritable obesity. The offspring of the other 11 pedigrees did not show obesity and high blood glucose. The F1 founder mouse of *Obese-13* weighed 47 g at the age of 10 weeks, which was the most severe occurrence of early-onset obesity among all mice screened. Similarly, the F1 founder mouse of *Obese-10* weighed 51 g at 18 weeks old and was the heaviest mouse at the age among all the mice screened, except for the F1 founder mouse of *Obese-13*. Thus, the two founders of the heritable obese pedigrees were two most obese mice out of the 5000 mice screened.

### Identification of the *Sim1* gene mutation in the *Obese-13* pedigree

**Figure 2:**
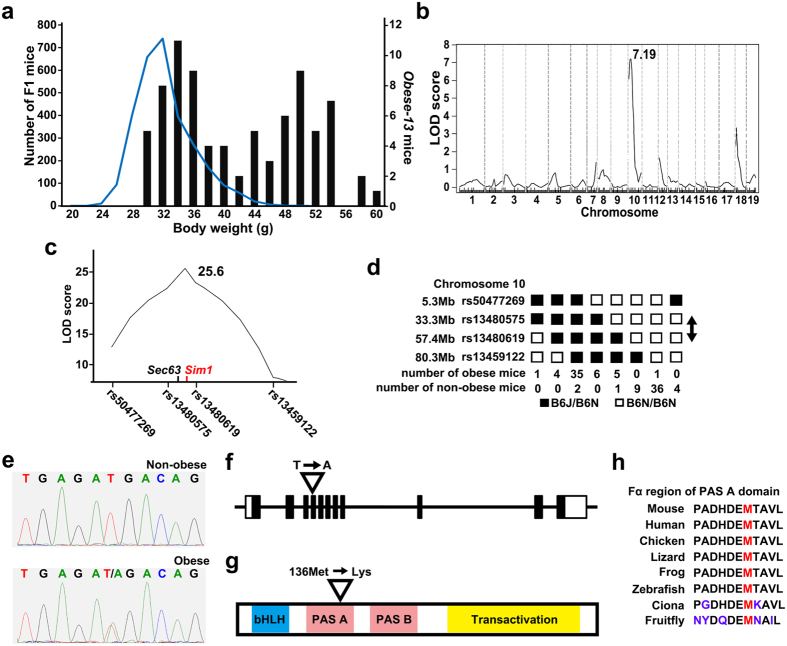
Identification of a single nucleotide substitution in the *Sim1* gene in the *Obese-13* pedigree. (**a**) The body weights of *Obese-13* pedigree N2 littermates (bars) are deviated toward overweight compared with the body weight distribution of the screened F1 mice (line, same as in Fig. 1b, Mann-Whitney U test, p < 0.0001). (**b**) QTL analysis of the *Obese-13* pedigree (n = 49) for body weight showed a single LOD score peak on chromosome 10. (**c**) Further QTL analysis using 172 N2 mice and focusing on chromosome 10 shows a single peak (LOD score, 25.6) located between rs13480575 and rs13480619. (**d**) Haplotype analysis of *Obese-13* N2 mice indicates that the mutation falls between rs13480575 and rs13480619 (arrow) on chromosome 10. (**e**) Direct sequencing of the *Sim1* gene identified a thymine (T)-to-adenine (A) substitution specific to obese mice of the *Obese-13* pedigree. (**f**) The T-to-A substitution is located in exon 4 of the *Sim1* gene. Black box indicates the coding region, while the white box indicates the non-coding region. (**g**) The methionine residue at position 136 located in the PAS A domain is substituted with lysine. (**h**) This methionine residue is well-conserved among vertebrates and invertebrates. The Fα region of the PAS A domain is indicated.

A body weight histogram of the *Obese-13* N2 male mice shows a deviation toward overweight relative to the body weight distribution of the F1 mice (Fig. 2a, Mann-Whitney U test, p < 0.0001). A linkage disequilibrium analysis of the *Obese-13* N2 mice (25 males, 24 females) identified a single and strong quantitative trait locus on chromosome 10 (Fig. 2b, LOD score 7.19). Further analysis using a larger number of the *Obese-13* N2 mice (male, n = 106; female, n = 66) and focusing on chromosome 10, showed a high LOD score (total:25.6, males: 22.8, females: 10.0) between rs13480575 (Chr10: 33372829) and rs13480619 (Chr10: 57472268) (Fig. 2c). A haplotype analysis of obese N2 mice (top 30 percent in body weight) and non-obese N2 mice (bottom 30 percent in body weight) is shown in Fig. 2d. Because random mutations were induced in B6J mice by the ENU treatment, obesogenic mutations should be closely linked to the B6J/N haplotype. Therefore, the haplotype analysis also supports the location of the mutation between rs13480575 (Chr10: 33372829) and rs13480619 (Chr10: 57472268). Among the 96 protein-coding genes located in this region, *Sim1* is the only gene that has been well characterized in relation to the pathogenesis of obesity in humans^22^,^23^,^24^ and rodents^25^,^26^,^27^,^28^,^29^. The direct sequencing of all 11 exons of the *Sim1* gene identified a single nucleotide substitution (Chr10: 50908536) of thymine to adenine in exon 4 specific to obese mice of the *Obese-13* pedigree (Fig. 2e,f). In parallel, we performed whole-exome sequencing of two severely obese mice (BW 62.5 g and 60.6 g at 26 weeks old) of the *Obese-13* pedigree and two obese mice of the *Obese-10* pedigree (Table 1). The two *Obese-13* obese mice had heterozygous non-synonymous mutations in the *Sim1* (Chr10: 50908536) and *Sec63* genes (Chr10: 42816394), whereas the two *Obese-10* obese mice did not. Both mutations were located within the mapped chromosomal region (Fig. 2c) and no other non-synonymous mutations were found on the chromosome 10. To further segregate these two mutations for the obese phenotype, we conducted direct sequencing of the *Sim1* and *Sec63* genes of five obese mice which had a chromosomal recombination between rs13480575 and rs13480619 (the fifth column in Fig. 2d) and of randomly selected five non-obese mice. All obese mice had mutations in both *Sim1* and *Sec63* genes, whereas all non-obese mice did not have any mutations in *Sim1* and *Sec63* genes. Thus, the mutations in the *Sim1* and *Sec63* genes were co-segregated due to its proximity. In one obese mouse that did not have the B6J/N haplotype (the second column from the last in Fig. 2d), we confirmed that the mouse did not have any mutations in the *Sim1* and *Sec63* genes, suggesting that obesity in this mouse may have been caused by the effects of other mutations or by other unknown factors. The mutation in the *Sec63* gene causes an isoleucine to methionine substitution at the residue 620. SEC63 is a part of a protein complex that translocates a nascent peptide into the endoplasmic reticulum and is associated with autosomal dominant polycystic liver disease^30^ (OMIM#608648). None of the obese mice of the *Obese-13* pedigree showed any cyst formations in the livers.

The identified mutation in the *Sim1* gene results in an amino acid substitution from methionine to lysine (M136K) in the PAS A domain of the SIM1 protein (Fig. 2g). SIM1 belongs to the basic helix–loop–helix-PER-ARNT-SIM (bHLH-PAS) transcription factor family^31^. The PAS A domain is composed of five helices and five strands^32^, and the substituted methionine is located in the Fα helix and is well conserved among mammals, birds, fishes and flies (Fig. 2h).

**Figure 3:**
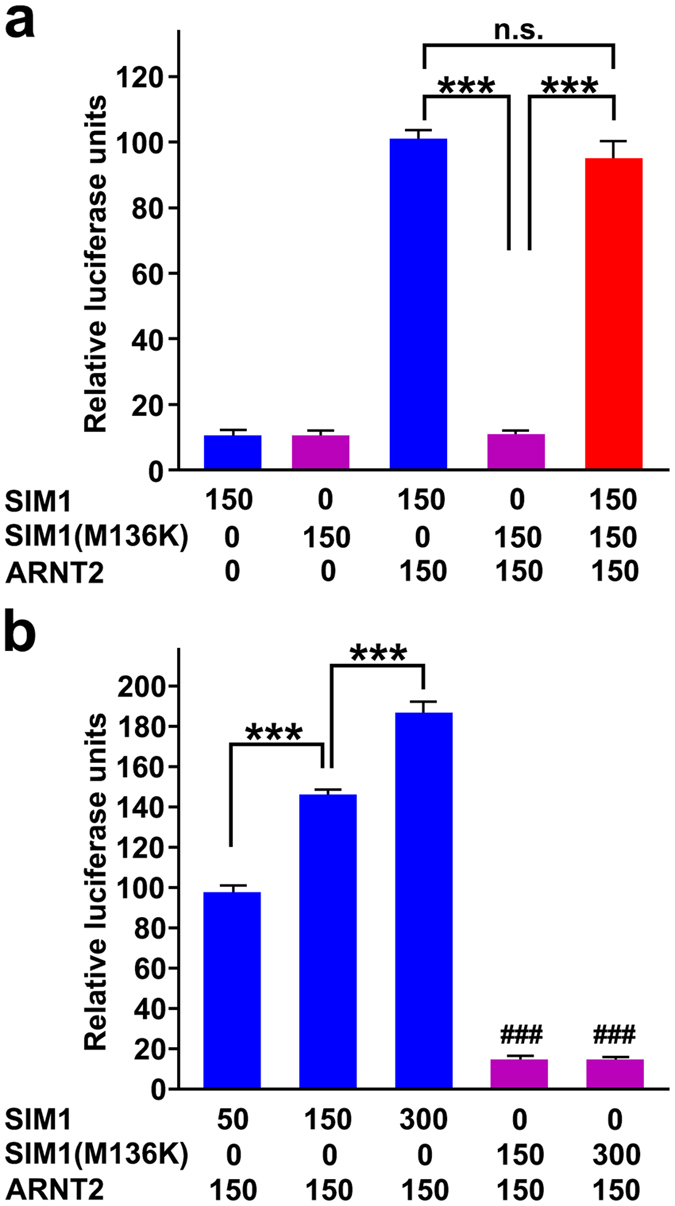
Luciferase assay of the SIM1 protein. (**a,b**) HEK293 cells were transiently transfected with wild type SIM1 and/or SIM1(M136K) expression plasmids, ARNT2 expression plasmids, SIM1 luciferase reporter plasmids and Renilla luciferase plasmids to assess transfection efficiency compensation. Bars indicate firefly/Renilla luciferase activity values relative to the mean firefly/Renilla values of the wild type SIM1 (**a**), 150 ng; (**b)**, 50 ng)- and ARNT2-transfected cells. ***p < 0.001. ^###^p < 0.001 indicates P values for comparisons between the SIM1 (M315K) protein group and any of the wild-type SIM1 protein groups. One-way ANOVA followed by Tukey’s post-hoc test. Data are shown as the mean + s.e.m.

Because SIM1 and other bHLH-PAS members form heterodimers with ARNT via the PAS domain to induce the transcription of target genes^31^,^32^, we assessed the effect of the M136K substitution on the transcriptional activity of SIM1:ARNT2 dimers using a luciferase reporter assay. Whereas the wild type SIM1 protein alone showed very low transcriptional activity, co-transfection with ARNT2 resulted in a high level of transcriptional activity, as reported previously (Fig. 3a)^33^. In contrast, co-transfection of SIM1(M136K) with ARNT2 failed to result in any transcriptional activity. We further examined whether SIM1(M136K) interferes with the activity of wild type SIM1 by transfecting SIM1, SIM1(M136K) and ARNT2 together. The presence of SIM1(M136K) did not alter the transcriptional activity of the SIM1:ARNT2 dimer (Fig. 3a), showing that SIM1(M136K) is devoid of a dominant negative effect on the wild-type SIM1 protein. When the SIM1 content was increased with the ARNT2 content remaining stable, luciferase units increased in a dose-dependent manner (Fig. 3b). However, increased amounts of SIM1(M136K) did not result in any increase in luciferase units.

### Metabolic characterization of *Sim1* ^*M136K*/+^ mice

**Figure 4:**
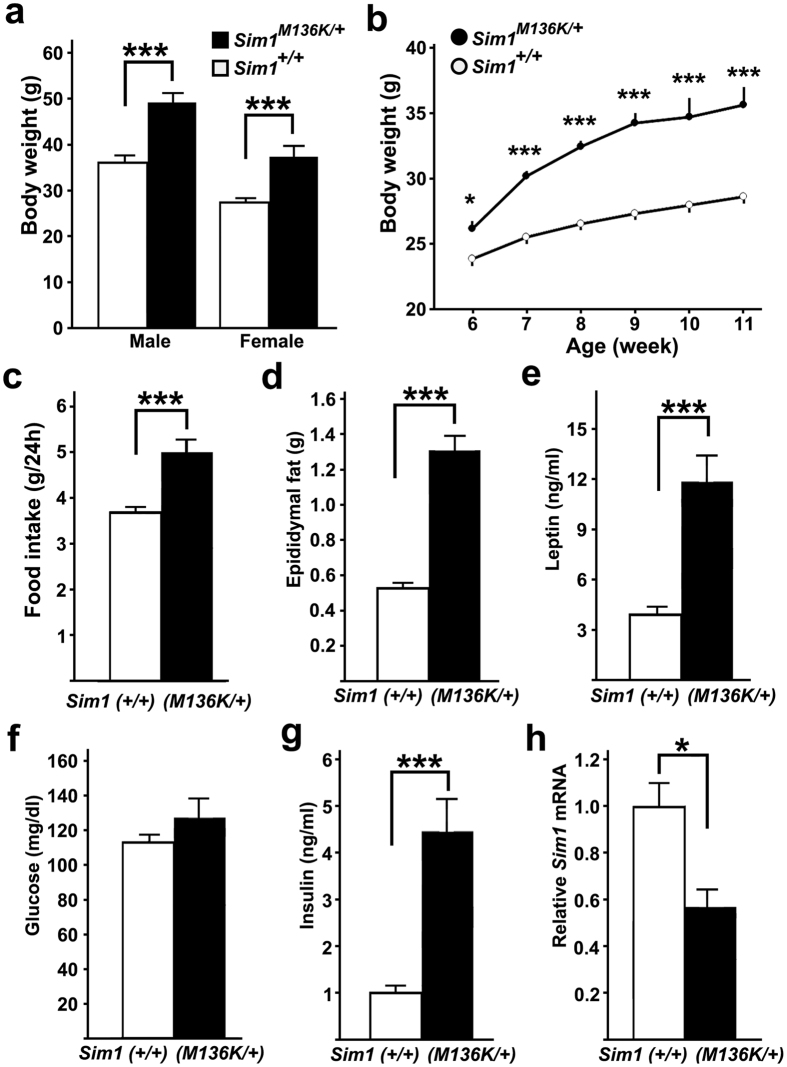
Metabolic phenotypes of *Sim1* mutant mice. (**a**) Both male and female *Sim1*^*M136K*/+^ mice (male n = 13, female n = 11) showed higher body weights than *Sim1*^+/+^ mice (male n = 12, female n = 13) at 26 weeks old in the N2 generation (Mann-Whitney U test, p < 0.0001). (**b**) Increased body weight of N3 mice indicates early-onset obesity in *Sim1*^*M136K*/+^ mice. *Sim1*^+/+^ mice, n = 19; *Sim1*^*M136K*/+^ mice, n = 10. *p < 0.05, ***p < 0.001, one-way repeated-measures ANOVA followed by Tukey’s post-hoc test. (**c**) Daily food intake of *Sim1*^*M136K*/+^ mice (n = 6) was higher than that of *Sim1*^+/+^ mice (n = 6) (**d**) Epididymal fat weights of *Sim1*^*M136K*/+^ mice (n = 6) and *Sim1*^+/+^ mice (n = 6). (**e**) Serum leptin levels of *Sim1*^*M136K*/+^ mice (n = 8) were higher than those of *Sim1*^+/+^ mice (n = 8). (**f**) Blood glucose levels were similar between *Sim1*^+/+^ mice (n = 19) and *Sim1*^*M136K*/+^ mice (n = 10). (**g**) *Sim1*^*M136K*/+^ (n = 8) mice had higher serum insulin levels than *Sim1*^+/+^ mice (n = 8). (**h**) *Sim1* mRNA levels of the medial hypothalamus of *Sim1*^*M136K*/+^ mice (n = 6) was lower than those of *Sim1*^+/+^ mice (n = 6). *p < 0.05, ***p < 0.001, two-tailed *t*-test. Data are shown as the mean + s.e.m.

Retrospective genotyping of N2 mice confirmed that the body weights of both male and female *Sim1*^*M136K*/+^ mice were significantly greater than those of *Sim1*^+/+^ mice (Fig. 4a). Further analysis of N3 mice revealed that the significant increase in the body weight of *Sim1*^*M136K*/+^ mice was present as early as 6 weeks of age (Fig. 4b). At the age of 9 weeks, *Sim1*^*M136K*/+^ mice had a significantly greater daily food intake than *Sim1*^+/+^ mice (Fig. 4c). The epididymal fat mass of *Sim1*^*M136K*/+^ mice was significantly larger than that of *Sim1*^+/+^ mice at the age of 12 weeks (Fig. 4d). Consistent with a larger fat mass, *Sim1*^*M136K*/+^ mice had higher serum leptin levels than *Sim1*^+/+^ mice (Fig. 4e). Although *Sim1*^*M136K*/+^ and *Sim1*^+/+^ mice had similar blood glucose levels at the late light phase in a fed condition, the serum insulin level in *Sim1*^*M136K*/+^ mice was significantly higher than that in *Sim1*^+/+^ mice (Fig. 4f,g). The *Sim1* mRNA content of the medial hypothalamus of *Sim1*^*M136K*/+^ mice was higher than that of *Sim1*^+/+^ mice (Fig. 4h).

### Identification of the *Mc4r* gene mutation in the *Obese-10* pedigree

**Figure 5:**
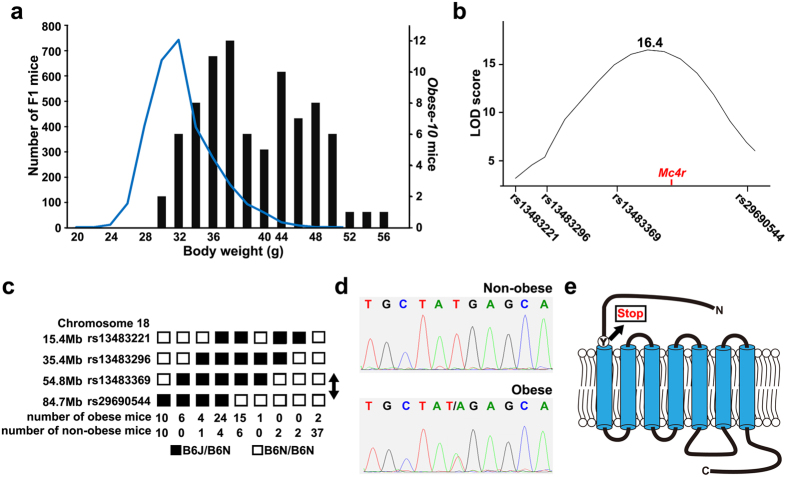
Identification of a single nucleotide substitution in the *Mc4r* gene in the *Obese-10* pedigree. (**a**) The body weights of *Obese-10* pedigree N2 littermates (bars) are deviated toward overweight compared with the body weight distribution of the screened F1 mice (line, same as in Fig. 2a). (**b**) QTL analysis of the *Obese-10* pedigree (n = 206) for body weight showed a single LOD score peak between rs13483369 and rs29690544 on chromosome 18. (**c**) Haplotype analysis of *Obese-10* N2 mice indicates that the mutation falls between rs13483369 and rs29690544 (arrow) on chromosome 18. (**d**) Direct sequencing of the *Mc4r* gene identified a thymine-to-adenine substitution specific to obese mice of the *Obese-10* pedigree. (**e**) The mutation results in a premature stop codon at the start of the first transmembrane domain of the MC4R, a G-protein coupled receptor.

Similar to the *Obese-13* pedigree, the body weight histogram of the *Obese-10* N2 male mice was clearly deviated toward overweight relative to the body weight distribution of the F1 mice (Fig. 5a, Mann-Whitney U test, p < 0.0001). A linkage disequilibrium analysis of the *Obese-10* N2 male mice (n = 23) identified a single quantitative trait locus on chromosome 18, with an LOD score of 3.94. Further analysis using a larger number of the *Obese-10* N2 mice (males, n = 107; females, n = 99) and focusing on the chromosome 18 showed a high LOD score (total: 16.4, male: 10.7, females: 13.3) between rs13483369 (Chr18: 54774495) and rs29690544 (Chr18: 84686237) (Fig. 5b). A haplotype analysis of obese N2 mice (top 30 percent in body weight) and non-obese N2 mice (bottom 30 percent in body weight) supports that an obesogenic mutation is located between rs13483369 (Chr18: 54774495) and rs29690544 (Chr18: 84686237) (Fig. 5c). Among the 147 protein-coding genes located in this region, the *Mc4r* gene has been well characterized in relation the pathogenesis of obesity in humans^34^,^35^,^36^,^37^ and rodents^38^,^39^. The OMIM database summarizes more than 20 heterozygous variants of the *MC4R* gene that are associated with human obesity (http://www.omim.org/entry/155541#0022). Direct sequencing of the entire *Mc4r* gene identified a single nucleotide substitution (Chr18: 66859918) of thymine to adenine specific to obese mice of the *Obese-10* pedigree (Fig. 5d). In parallel, whole-exome sequencing of two obese mice (BW 56.3 g and 54.5 g at 26 weeks old) of the *Obese-10* pedigree confirmed that the thymine to adenine substitution in the *Mc4r* gene (Chr18: 66859918) was the only non-synonymous nucleotide change that is located within the chromosome 18 region linked to obese phenotype (Table 1, Fig. 5b). Obese mice of the *Obese-13* pedigree did not have the *Mc4r* gene mutation. Thus, the mutation of the *Mc4r* gene is thought to be responsible for obesity in the *Obese-10* pedigree. The identified mutation in the *Mc4r* gene results in a premature stop codon at the beginning of the first transmembrane domain of the MC4R protein (Y41X) (Fig. 5e). In addition, we confirmed the presence of the wild type *Mc4r* gene in the two obese mice that did not have the B6J/N haplotype (the last column in Fig. 5d), suggesting that obesity in these mice may have been of other mutations or other unknown factors.

### Metabolic characterization of *Mc4r* ^*Y41X*/+^ mice

**Figure 6:**
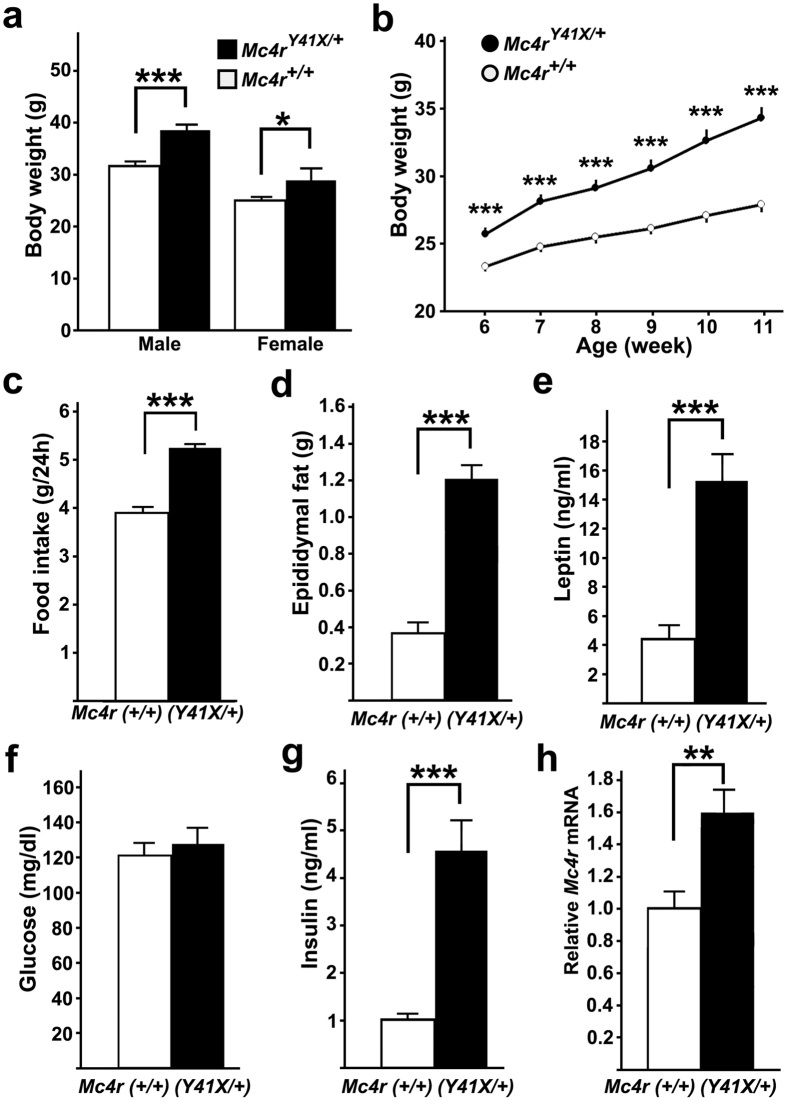
Metabolic phenotypes of *Mc4r* mutant mice. (**a**) Both male and female *Mc4r*^*Y41X*/+^ mice (male n = 11, female n = 4) showed higher body weights than *Mc4r*^+/+^ mice (male n = 11, female n = 8) at 26 weeks old in the N2 generation. (**b**) Increased body weight of N3 mice indicates early-onset obesity in *Mc4r*^*Y41X*/+^ mice (n = 16) relative to *Mc4r*^+/+^ (n = 16) mice. ***p < 0.001, one-way repeated-measures ANOVA followed by Tukey’s post-hoc test. (**c**) Daily food intake of *Mc4r*^*Y41X*/+^ mice (n = 6) was higher than that of *Mc4r*^+/+^ mice (n = 6) (**d**) Epididymal fat weights of *Mc4r*^*Y41X*/+^ mice (n = 6) and *Mc4r*^+/+^ mice (n = 6). (**e**) Serum leptin levels of *Mc4r*^*Y41X*/+^ mice (n = 8) were higher than those of *Mc4r*^+/+^ mice (n = 8). (**f**) Blood glucose levels were similar between *Mc4r*^+/+^ mice (n = 16) and *Mc4r*^*Y41X*/+^ mice (n = 16). (**g**) *Mc4r*^*Y41X*/+^ mice (n = 8) had higher serum insulin levels than *Mc4r*^+/+^ mice (n = 8). (**h**) *Mc4r* mRNA levels of the medial hypothalamus of *Mc4r*^*Y41X*/+^ mice (n = 8) was higher than those of *Mc4r*^+/+^ mice (n = 6). *p < 0.05, **p < 0.01, ***p < 0.001, two-tailed *t*-test. Data are shown as the mean + s.e.m.

Retrospective genotyping of N2 mice confirmed that the body weights of both male and female *Mc4r*^*Y41X*/+^ mice were significantly greater than those of *Mc4r*^+/+^ mice (Fig. 6a). Further analysis of N3 mice revealed that the significant increase in the body weight of *Mc4r*^*Y41X*/+^ mice was found as early as 6 weeks of age and that the difference persisted with increasing age (Fig. 6b). *Mc4r*^*Y41X*/+^ mice had a significantly greater daily food intake than *Mc4r*^+/+^ mice (Fig. 6c). The epididymal fat mass of *Mc4r*^*Y41X*/+^ mice was significantly larger than that of *Mc4r*^+/+^ mice at the age of 12 weeks (Fig. 6d). Consistent with a larger fat mass, *Mc4r*^*Y41X*/+^ mice had higher serum leptin levels than *Mc4r*^+/+^ mice (Fig. 6e). Although *Mc4r*^*Y41X*/+^ and *Mc4r*^+/+^ mice had similar blood glucose levels at the late light phase in a fed condition, the serum insulin level of *Mc4r*^*Y41X*/+^ mice was significantly higher than that of *Mc4r*^+/+^ mice (Fig. 6f,g). Interestingly, the *Mc4r* mRNA level of the medial hypothalamus of *Mc4r*^*Y41X*/+^ mice was higher than that of *Mc4r*^+/+^ mice (Fig. 6h).

### Altered hypothalamic neuropeptide gene expression in *Sim1* ^*M136K*/+^ mice, *Mc4r* ^*Y41X*/+^ mice and mice with diet-induced obesity

**Figure 7:**
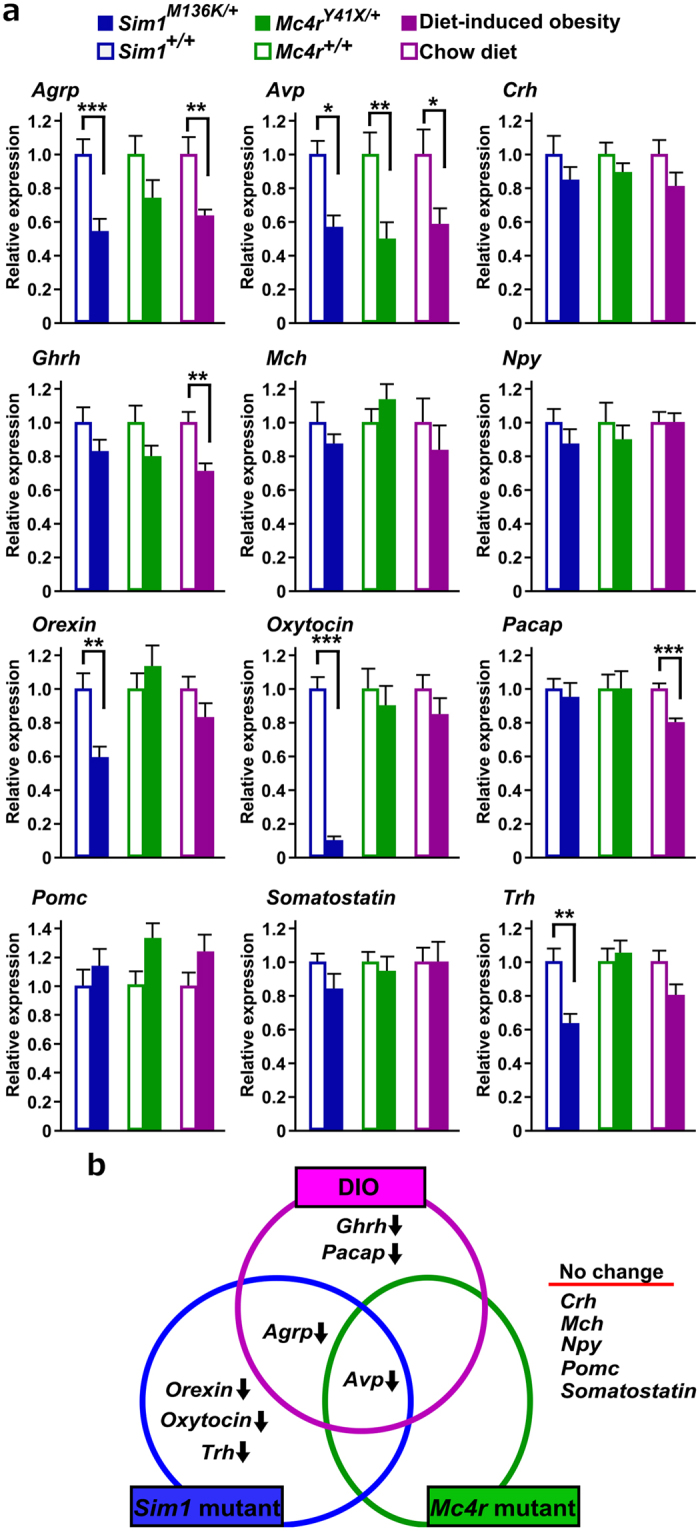
Hypothalamic neuropeptide gene expression in three mouse models of obesity. (**a**) Quantitation of hypothalamic neuropeptide gene expression in *Sim1*^+/+^ (n = 9), *Sim1*^*M136K*/+^ (n = 9), *Mc4r*^+/+^ (n = 9), *Mc4r*^*Y41X*/+^ (n = 9), and high-fat-diet-induced obesity (DIO) (n = 9) mice and in mice fed a regular chow diet (n = 9). Values shown are expressed relative to the average of the control groups for each comparison. (**b**) Altered profiles of neuropeptide gene expression of the three models of mouse obesity. *p < 0.05, **p < 0.01, ***p < 0.001, two-tailed *t-*test with Bonferroni correction for multiple comparisons. Data are shown as the mean + s.e.m.

Since, in the medial hypothalamus, SIM1 is expressed only in the PVN and the knockdown of *Sim1* in the PVN results in hyperphagia^40^, *Sim1*^*M136K*/+^ mice may have altered hypothalamic gene expressions that are associated with obesity. Similarly, the hypothalamic gene expressions of *Mc4r*^*Y41X*/+^ mice may be altered because MC4R is a direct target of NPY/AGRP and POMC neurons of the arcuate nucleus^4^. Previously, diet-induced obesity (DIO) in C57BL/6, a good model for human obesity, showed neuropeptide gene expression changes in the hypothalamus^8^,^41^. Then, we examined whether these three different models of obesity showed any similarities in hypothalamic gene expression changes. DIO mice and control mice were fed a high-fat diet and a regular chow diet, respectively, for 9 weeks, starting at the age of 3 weeks (chow mice, n = 9, body weight 24.7 ± 0.3 g; DIO mice, n = 9, body weight 30.2 ± 0.4 g). The mRNAs examined were for the following genes: *Agrp, Avp*, *Corticotrophin releasing hormone (Crh)*, *Growth hormone releasing hormone (Ghrh), Melanin concentrating hormone (Mch), Neuropeptide Y (Npy), Orexin, Oxytocin, Pituitary adenylate cyclase-activating polypeptide (Pacap), Pomc, Somatostatin* and *Thyrotropin releasing hormone (Trh)*. All three obese groups showed a reduced expression of *Avp* mRNA, whereas they did not show any changes in *Crh, Mch*, *Npy, Pomc,* or *Somatostatin* mRNA levels (Fig. 7a,b). Both *Sim1*^*M136K*/+^ and DIO mice showed a reduced expression of *Agrp* mRNAs. A significant reduction in *Orexin*, *Oxytocin* and *Trh* mRNAs was found in *Sim1*^*M136K*/+^ mice. In particular, *Sim1*^*M136K*/+^ mice showed a 90% reduction in *Oxytocin* mRNA. Decreases in *Ghrh* and *Pacap* mRNAs were identified only in DIO mice (Fig. 7a,b).

## Discussion

The current dominant screening for obesity identified single nucleotide substitutions in the *Sim1* and *Mc4r* genes of obese pedigrees. The mutation in the *Sim1* gene produces SIM1(M136K), which totally lacks transcriptional activity, and the mutation in the *Mc4r* gene of *Mc4r*^*Y41X*/+^ mice produces a premature stop codon at the first transmembrane domain of the seven transmembrane domains of this G protein-coupled receptor. Thus, *Sim1*^*M136K*/+^ and *Mc4r*^*Y41X*/+^ mice are actually heterozygous *Sim1*-deficient and *Mc4r*-deficient mice, respectively, and thus exhibited overweight and increased leptin and insulin levels, as found in heterozygous *Sim1*- or *Mc4r*-deficient mice^27^,^28^,^29^,^38^. To the best of our knowledge, *Sim1* and *Mc4r* genes are the only genes that cause severe obesity via genetic haploinsufficiency. Although heterozygous deficiencies in brain-derived neurotrophic factor (BDNF)^42^ also cause obesity, the body weight of these adult heterozygous mutant mice is approximately 35 g, which is much lower than the threshold we set for our determination of obese phenodeviants. Recessive screening for obesity is necessary to isolate additional genes that are involved in energy metabolism.

In addition to the *Sim1* mutation, obese mice of the *Obese-13* pedigree had a non-synonymous, heterozygous mutation in the *Sec63* gene. SEC63 makes a complex with SEC61 and SEC62 to transport a newly synthesized peptide into the endoplasmic reticulum^30^. In humans, SEC63 mutations are associated with autosomal dominant polycystic liver disease^30^,^43^ (OMIM#608648). Consistently, loss of SEC63 in kidneys causes cyst formations^44^. However, none of the obese mice of the *Obese-13* pedigree showed any cyst formations. As for metabolism, any human SNPs around the *SEC63* gene have not been related to obesity, adiposity and diabetes according to the GWAS Central data base (http://www.gwascentral.org/phenotypes). We did not find any literature showing any mechanistic or genetic links between SEC63 and obesity. Heterozygous SEC63-deficient mice were viable and fertile^44^, but obese mice of the *Obese-13* pedigree were almost infertile. Although we cannot deny the possibility of the heterozygous mutation in the *Sec63* gene can exert any effect on overweight, there have been no evidence support this possibility.

This first successful dominant obesity screening illustrates the advantage of the combined use of B6 substrains as mutagenized and counter strains, *in vitro* fertilization for the production of next generation and whole-exome sequencing to identify mutations within the chromosomal region that is linked to the target phenotype. In general, the metabolic similarity between the mutagenized and counter strains makes the screening more sensitive and reproducible. We did not find any significant differences in QTLs between B6J and B6N in relation to body weight, which is consistent with their common origin and genetic similarity^18^. Based on published mutation rates and the number of non-synonymous mutations^11^,^45^, offspring of mice treated with ENU have 50–70 mutations. Consistent with this number, whole-exome sequencing showed that N2 generation mice of the *Obese-10* and *Obese-13* pedigrees had 20–40 non-synonymous mutations (Table 1). If a linkage analysis succeeds in identifying a chromosomal region linked to a target phenotype, the mapped chromosomal region usually contains one or two mutations, as is the case with the *Obese-10* and *Obese-13* pedigrees.

The routine use of *in vitro* fertilization is required for the production of offspring when the target phenotype is accompanied by low fertility. Mice with severe obesity are generally infertile^20^,^21^, and in fact, obese male and female mice of the *Obese-10* and *Obese-13* pedigrees were not able to have offspring via natural mating. *In vitro* fertilization overcomes the high risk of discontinuity of obese pedigrees and the inability to assess the heritability of obese phenotypes due to a lack of offspring.

Although we selected the 13 most obese mice to examine the heritability of obesity, only two pedigrees showed heritable obesity. The other 11 mice may have been overweighed due to the combined effects of weak obesogenic mutations or because of random fluctuations in body weight. Because the distribution of body weight tends to skew toward overweight, as shown in Fig. 1b, it is more likely that many obese mice are found by chance. As Takahashi *et al*. discussed^15^, if the phenotypic effect of a mutation is not strong enough, it is not possible to classify individual mice as either wild type or mutant. Actually, the founders of *Obese-10* and *Obese-13* were the two heaviest mice of the 5000 mice that were screened. In other words, it is very challenging to detect gene mutations through a dominant screening that cause weak to moderate obesity.

Interestingly, the changes in hypothalamic neuropeptide expression in *Sim1*^*M136K*/+^ mice and *Mc4r*^*R41X*/+^ mice only partially overlap despite the very similar patterns of energy and glucose metabolism in both obese mutant pedigrees. Because SIM1 is a transcription factor strongly expressed in the PVN and is required for the production of AVP and oxytocin and the development of TRH neurons^25^, a SIM1 haploinsufficiency may directly increase or decrease the transcription levels of target genes. In fact, we found that *Sim1*^*M136K*/+^ mice displayed a 90% reduction in *oxytocin* mRNA level and significant reductions in *Avp* and *Trh* mRNA levels, which is consistent with observations in heterozygous *Sim1*-deficient mice^46^. In contrast, orexin is expressed in the LHA, which is devoid of SIM1, indicating that the reduced expression of *orexin* mRNA in *Sim1*^*M136K*/+^ mice is not a direct consequence of the disrupted transcriptional activity of the mutant SIM1 protein but instead a secondary change based on the tight reciprocal fiber connections between the PVN and orexin neurons^47^. Because orexin works to suppress overweight gains^7^,^8^, decreased *orexin* expression may promote overweight.

In contrast to *Sim1*, *Mc4r* is expressed in a number of brain regions, including the arcuate nucleus, dorsomedial nucleus, PVN, the parabrachial nucleus and the solitary tract nucleus^3^,^48^. The lack of significant changes in neuropeptide expression in the hypothalamus of *Mc4r*^*R41X*/+^ mice, other than the change in *Avp* mRNA level, implies that MC4R haploinsufficiency may cause obesity through mild effects at the multiple sites or in a transcription-independent manner. Unlike obesity in *Sim1* or *Mc4r* mutant mice, the obesity induced in the high-fat diet rodent model is relevant to human obesity which is strongly associated with the recent availability of high-calorie food. Prolonged high-fat diet feeding promotes leptin-resistance^1^,^8^ and alters hypothalamic gene expression patterns, such as decreased *Agrp* and *Ghrh* mRNA levels without significant change in *Pomc* mRNA level^41^,^49^,^50^,^51^, which is consistent with the current results for DIO mice. Common changes between DIO mice and genetically obese mice, such as decreased *Agrp* and *Avp* mRNA levels, might be secondary to obesity-associated changes, including increased leptin and insulin levels and polyuria^52^. Furthermore, the altered expressions of *Ghrh* and *Pacap* mRNAs in DIO mice may be associated with behavioral changes and not related to metabolic changes because eating a high-fat diet induces behavioral changes in addition to obesity^53^. Thus, despite the involvement of the hypothalamus in obesity pathogenesis of three obese models, each model has a distinct pattern of hypothalamic gene expressions.

Because there has been a rich accumulation of data on the genetics of obesity in rodents and humans^54^,^55^, we were able to select the *Sim1* and *Mc4r* genes as candidate genes. However, this candidate gene approach would not work in fields such as sleep research in which very few of the genes involved have been identified and instead whole-exome sequencing is necessary for the mutation search. In conclusion, the combined use of B6 substrains, *in vitro* fertilization and whole-exome sequencing is very versatile and is applicable to any research areas to identify genes that have a major effect on a target behaviors and physiological parameters.

## Methods

### Animals and mutagenesis

Male C57BL/6J mice (CLEA Japan) were treated with ethylnitrosourea (85 mg/kg BW, Sigma-Aldrich) via intraperitoneal injections twice at weekly intervals at 8 weeks old^17^. At the age of 25–30 weeks, the sperm of the mice were collected and used for the *in vitro* fertilization of eggs from C57BL/6N mice to obtain F1 offspring. Mice were provided food and water ad libitum, maintained on a 12-hour light/dark cycle and housed under controlled temperature and humidity conditions. All procedures were carried out in accordance with the Guidelines for Animal Experiments of the University of Tsukuba and the RIKEN Tsukuba Institute and were approved by the Institutional Animal Care and Use Committee of the University of Tsukuba and the RIKEN Tsukuba Institute.

### Screening scheme for heritable obesity

The screening for obesity was performed in parallel with examining the mice for sleep/wakefulness abnormalities using the same randomly mutagenized group of mice. At the age of 12 weeks, F1/G1 male mice had an EEG/EMG electrode implanted while under isoflurane anesthesia and were then examined for sleep/wakefulness abnormalities. The sleep study was performed for F1/G1 male EEG, and not for their offspring. Thirteen obese mice were selected to produce progeny via *in vitro* fertilization of eggs from C57BL/6N mice. When at least 30% of the progeny showed obesity similar to that of the founder mouse, we determined that the obesity phenotype was heritable. N2 generation mice were used for a linkage analysis. Body weights were measured weekly from the age of 10 weeks to 26 weeks, and biweekly from 28 weeks to 34 weeks. Blood glucose levels were determined at the late light phase under a fed condition^16^.

### Linkage analysis

Genomic DNA was purified from tail samples of N2 progeny using a DNeasy Blood & Tissue kit (Qiagen). SNPs of each N2 mouse were determined using a custom TaqMan Genotyping assay (Thermo Fisher) on an Applied Biosystems 7900HT Fast Real-Time PCR system controlled by SDS2.2.2 software and a TaqMan Genotyper (Thermo Fisher). The custom probes were designed based on the polymorphisms between C57BL/6J and C57BL/6N mice^19^. A QTL analysis was performed using J/qtl software (Jackson Laboratory).

### Whole-exome sequencing

Exome sequencing libraries were prepared with SeqCap EZ Developer Library kit (MM9 exome, cat# 110624, Roche NimbleGen) from 500 ng of genomic DNA. Sequencing of multiplexed library with 151 × 2 paired-end mode in NextSeq 500 sequencer (Illumina) was performed by i-Laboratory LLP sequencing platform (Tsukuba, Japan). Reads in FASTQ files were imported to CLC Genomics Workbench (Qiagen), trimmed by 1-base at 3′ end, and mapped to the mm10 mouse reference which represents C57BL/6J strain genome. Exome sequencing statistics was calculated for capture target region definition provided by SeqCap kit. Variant call was performed using Basic Variant Detection tool. Potential variants were filtered against control mice variants. Nonsynonymous base substitutions and small InDels were identified based on ENSEMBLE transcript annotation. All of variants identified on chromosomes 10 and 18 for each pedigree were verified by visual inspection of sequence tag alignment. The numbers of the total reads and of variants called of four obese mice from the *Obese-10* and *Obese-13* pedigrees were summarized in Table 1.

### Mutation analysis and genotyping of *Mc4r* and *Sim1* mutant mice

PCR products amplified from genomic DNA were purified using a FastGene Gel/PCR extraction kit (Nippon Genetics). The sequencing reaction was performed using the Big Dye Terminator (v3.1) Cycle Sequencing kit followed by capillary electrophoresis on an ABI3130 Genetic Analyzer (Thermo Fisher). To detect the *Mc4r* gene mutation, genomic DNA was amplified using Mc4r S1 (5′-TCCACCCGGCTGACCACGATGGGA-3′) and Mc4r AS2 (5′-GGCATCCGTATCCGTACTGT-3′) and then digested with *Dde*I, which recognizes only the mutant Mc4r sequence. Electrophoresis on 2% gel showed a 353 bp band for the wild type gene and 219 bp and 134 bp bands for the Mc4r mutant gene. The single nucleotide variant of the *Sim1* gene was also detected via the derived cleaved and amplified polymorphic sequence (dCAPS) method using primers designed with dCAPS Finder 2.0 (http://helix.wustl.edu/dcaps/dcaps.html). Genomic DNA was amplified using Sim1 dCAPS S1 (5′-TCCACCCGGCTGACCACGATGGGA-3′) and Sim1 dCAPS AS2 (5′-AAGGCTCAGGAGGAGAGAGG-3′) to produce a *Fok*I recognition sequence, GGATG, specific to the wild type *Sim1* sequence. PCR products were digested with *Fok*I and then separated via electrophoresis on 2.5% agarose gel.

### SIM1 luciferase assay

Full-length mouse *Sim1* and *Arnt2* cDNAs were amplified from mouse hypothalamic cDNA, which was reverse-transcribed from mRNA prepared using a PrimeScript RT kit (Takara). *Sim1* cDNA and *Arnt2* cDNA were subcloned into pcDNA3.1 vectors using an In-Fusion HD Cloning kit (Takara-Bio). A single nucleotide substitution was introduced into *Sim1* cDNA using a KOD-Plus-Mutagenesis kit (Toyobo #SMK-101) to express SIM1(M143K). The reporter plasmid was constructed by inserting a 6x CNS midline enhancer (CME) sequence of the Drosophila *toll4* gene^56^ and a TATA box into the upstream region of the firefly luciferase gene of the pGL3-basic vector (Promega) as previously described for pML/6C-WT vector^33^. HEK293 cells were maintained in DMEM supplemented with 10% fetal bovine serum and penicillin/streptomycin. Cells were plated in 24-well plates at a density of 50,000 cells per well. Twenty-four hours later, cells were transiently transfected with pcDNA3-*Sim1* (50, 150, 300 ng) or pcDNA3-*Sim1(M143K)* (150, 300 ng) and pcDNA3-*Arnt2* (150 ng), pML-6xCME-Luc (150 ng), and pRL-SV40 (40 ng) vectors per well using Lipofectamine LTX (Life Technologies). The total amount of transfected plasmids was adjusted to 640 ng by adding appropriate amounts of the pcDNA3 plasmid. Forty-eight hours post-transfection, cells were assayed for luciferase activity using a Dual-Luciferase Reporter Assay System (Promega). Each assay was performed in triplicate and was repeated three times. Firefly luciferase activity was normalized to Renilla luciferase activity for each well.

### Measurement of daily food intake

The mice were individually housed and fed powered chow (MF; Oriental Yeast) in a small food jar. After one week of habituation to the powdered food, the amount of food intake was measured at every 24 hours for seven consecutive days.

### Diet-induced obesity mice

C57BL/6 mice were fed a high-fat diet (D12492; Research Diet) starting at 3 weeks of age. The low-fat diet, or normal chow, (MF; Oriental Yeast) provided 3.6 kcal/g (61% carbohydrate, 26% protein, and 13% fat), whereas the high-fat diet provided 5.2 kcal/g (20% carbohydrate, 20% protein, and 60% fat). After 9 weeks of being fed the high-fat diet, mice were sacrificed at ZT11.

### Blood analysis

Blood glucose was measured from tail blood using Glutest kits (Sanwa Kagaku) at the late light phase in a fed condition. To measure insulin and leptin levels, blood was collected from the tail vein or orbital sinus of mice under anesthesia at the late light phase in a fed condition. The blood was centrifuged, and the serum was then stored at −80 °C until use. Samples were analyzed using Mouse Insulin ELISA kits and Mouse Leptin ELISA kits (Morinaga Institute of Biological Science, Yokohama, Japan).

### Tissue preparation

Mice were sacrificed via cervical dislocation while deeply anesthetized with sodium pentobarbital (50 mg/kg body weight). Then, the brain was rapidly removed, and the medial hypothalamus was dissected on ice based on the following boundaries: rostral, the optic chiasm; caudal, the mammillary bodies; 1 mm bilateral from the midline; and 1.5 mm dorsal of the ventral surface. This dissected tissue included the arcuate nucleus, ventromedial hypothalamic nucleus, dorsomedial hypothalamic nucleus, paraventricular hypothalamic nucleus, anterior hypothalamic area, and the medial half of the lateral hypothalamic area. At the same time, epididymal fat tissues were removed and weighed.

### Quantitative RT-PCR

Total RNA was isolated using the RNeasy Lipid Tissue Mini kit (Qiagen) and used for cDNA synthesis with oligo dT primers and a PrimeScript reverse transcriptase kit (TaKaRa). Real-time quantitative PCR reactions were performed with ViiA7 Real-Time PCR System (ThermoFisher) using SYBR GREEN PreMix Ex Taq (TaKaRa). The following PCR primers were used: *Agrp* forward, 5′-TCCCAGAGTTCCCAGGTCTA-3′; *Agrp* reverse, 5′-GCCAAAGCTTCTGCCTTCT-3′; *Avp* forward, 5′-AGGATGCTCAACACTACGCTCT -3′; *Avp* reverse, 5′-ACTGTCTCAGCTCCATGTCAGA -3′; *Crh* forward, 5′-GAAAGGGAAAAGGCAAAAGAA-3′; *Crh* reverse, 5′-GTTAGGGGCGCTCTCTTCTC-3′; *Gapdh* forward, 5′-AGAACATCATCCCTGCATCC-3′; *Gapdh* reverse, 5′-CACATTGGGGGTAGGAACAC-3′; *Ghrh* forward, 5′-CTCTTTGTGATCCTCATCCTCAC-3′; *Ghrh* reverse, 5′-AGTTTCCTGTAGTTGGTGGTGAA-3′; *Mc4r* forward, 5′-GCCAGGGTACCAACATGAAG-3′; *Mc4r* reverse, 5′-ATGAAGCACACGCAGTATGG-3′; *Mch* forward, 5′-TGCTGAGTCCACACAGGAAA-3′; *Mch* reverse, 5′-GCCAACATGGTCGGTAGACT-3′; *Npy* forward, 5′-TACTCCGCTCTGCGACACTA-3′; *Npy* reverse, 5′-TCACCACATGGAAGGGTCTT-3′; *Orexin* forward, 5′-GGGTATTTGGACCACTGCAC-3′; *Orexin* reverse, 5′-CCCAGGGAACCTTTGTAGAAG-3′; *Oxytocin* forward, 5′-GCCAGGAGGAGAACTACCTG-3′; *Oxytocin* reverse, 5′-CTCCGAGAAGGCAGACTCAG-3′; *Pacap* forward, 5′-CTATGGCTATTGCTATGCACTCTG-3′; *Pacap* reverse, 5′- CAACCTGGGGAAGACTCATTTAG-3′; *Pomc* forward, 5′-AACCTGCTGGCTTGCATC-3′; Pomc reverse, 5′-TTTTCAGTCAGGGGCTGTTC-3′; *Sim1* forward, 5′-CCTCCATCCACAGAATCCAC-3′; *Sim1* reverse, 5′-TGATACTGTTCGGTGCGGTA-3′; *Somatostatin* forward, 5′-CTCTGCATCGTCCTGGCTTT-3′; *Somatostatin* reverse, 5′-AAGTACTTGGCCAGTTCCTGTTT-3′; *Trh* forward, 5′-GAAGGTGCTGTGACTCCTGAC-3′; *Trh* reverse, 5′-ATCTAAGGCAGCACCAAGGTC-3′.

A relative quantification method was employed for the quantification of target molecules, which calculated the ratio between the amount of the target molecule and a reference molecule within the same sample, according to the manufacturer’s protocol. The reactions were performed in duplicate and the results were averaged. The averages of *glyceraldehyde-3-phosphate dehydrogenase (GAPDH)* mRNA were used for normalization.

### Statistics

Sample sizes were determined using R software based on the averages and standard deviations that were obtained from small scale experiments. The experimenters were blinded to genotypes and treatment assignments. Statistical analyses were performed using SPSS Statistics 22 (IBM) and R software. All data were tested for Gaussian distribution and variance. Homogeneity of variance was tested for using Levene’s test. P < 0.05 was considered statistically significant.

## Additional Information

**How to cite this article**: Hossain, M. S. *et al*. Identification of mutations through dominant screening for obesity using C57BL/6 substrains. *Sci. Rep.* **6**, 32453; doi: 10.1038/srep32453 (2016).

## Figures and Tables

**Table 1:** Whole-exome sequencing identifies non-synonymous mutations within the mapped chromosomal regions.

Mouse ID	Obese-10 A		Obese-10 B		Obese-13 A		Obese-13 B	
Body weight (26 weeks)	56.3 g		54.5 g		62.5 g		60.6 g	
Total reads	85,845,404	100.0%	74,370,332	100.0%	78,108,258	100.0%	84,534,362	100.0%
Mapped reads	76,972,358	89.7%	66,530,500	89.5%	68,763,865	88.0%	73,595,915	87.1%
Not mapped reads	8,873,046	10.3%	7,839,832	10.5%	9,344,393	12.0%	10,938,447	12.9%
Average coverage of capture target regions (x)	93		80		81		86	
Minimum coverage of capture target regions								
5x		97.2%		96.1%		96.3%		96.8%
10 x		93.4%		90.7%		91.0%		92.3%
20 x		81.3%		75.2%		75.8%		78.7%
40 x		54.3%		46.4%		47.0%		50.3%
80 x		25.6%		21.6%		22.0%		23.4%
100 x		19.9%		17.1%		17.4%		18.3%
Variants called	8,693	100.0%	7,393	100.0%	7,453	100.0%	7,818	100.0%
After filtering against control	626	7.2%	457	6.2%	443	5.9%	535	6.8%
With amino acid change	40	0.5%	31	0.4%	21	0.3%	29	0.4%
Sec63 (chr10: 42816394)	A/A		A/A		A/G		A/G	
Sim1 (chr10: 50908536)	T/T		T/T		T/A		T/A	
Mc4r(chr18:66859918)	A/T		A/T		A/A		A/A	
